# Bile diversion, a bariatric surgery, and bile acid signaling reduce central cocaine reward

**DOI:** 10.1371/journal.pbio.2006682

**Published:** 2018-07-26

**Authors:** India A. Reddy, Nicholas K. Smith, Kevin Erreger, Dipanwita Ghose, Christine Saunders, Daniel J. Foster, Brandon Turner, Amanda Poe, Vance L. Albaugh, Owen McGuinness, Troy A. Hackett, Brad A. Grueter, Naji N. Abumrad, Charles Robb Flynn, Aurelio Galli

**Affiliations:** 1 Neuroscience Program, Vanderbilt University School of Medicine, Nashville, Tennessee, United States of America; 2 Department of Molecular Physiology and Biophysics, Vanderbilt University Medical Center, Nashville, Tennessee, United States of America; 3 Department of Anesthesiology, Vanderbilt University Medical Center, Nashville, Tennessee, United States of America; 4 Department of Pharmacology, Vanderbilt University Medical Center, Nashville, Tennessee, United States of America; 5 Department of Surgery, Vanderbilt University Medical Center, Nashville, Tennessee, United States of America; 6 Department of Hearing and Speech Sciences, Vanderbilt University Medical Center, Nashville, Tennessee, United States of America; 7 Department of Surgery, University of Alabama at Birmingham, Birmingham, Alabama, United States of America; University of Pennsylvania Perelman School of Medicine, United States of America

## Abstract

The gut-to-brain axis exhibits significant control over motivated behavior. However, mechanisms supporting this communication are poorly understood. We reveal that a gut-based bariatric surgery chronically elevates systemic bile acids and attenuates cocaine-induced elevations in accumbal dopamine. Notably, this surgery reduces reward-related behavior and psychomotor sensitization to cocaine. Utilizing a knockout mouse model, we have determined that a main mediator of these post-operative effects is the Takeda G protein-coupled bile acid receptor (TGR5). Viral restoration of TGR5 in the nucleus accumbens of TGR5 knockout animals is sufficient to restore cocaine reward, centrally localizing this TGR5-mediated modulation. These findings define TGR5 and bile acid signaling as pharmacological targets for the treatment of cocaine abuse and reveal a novel mechanism of gut-to-brain communication.

## Author summary

Communication between the gut and the brain is increasingly being appreciated as influencing motivated behavior. The gut can influence brain function through secreted hormones traveling through the blood and entering the brain. We utilize a weight-loss surgery designed to elevate one class of circulating hormones, bile acids, to show their action in the brain and their role in modulating behaviors associated with the addictive properties of cocaine. This surgery reduces the reward-related behavior and the psychomotor effects of cocaine. Furthermore, we utilize a knockout mouse model to reveal that a specific bile acid receptor mediates some of the effects of bile acids over motivated behavior. Viral intervention studies localize this effect to a receptor population within the nucleus accumbens, a brain region central to the processing of reward. These findings identify a role for bile acids in blunting cocaine’s ability to alter brain function, generating novel and exciting directions for the treatment of cocaine abuse.

## Introduction

**Fig 1:**
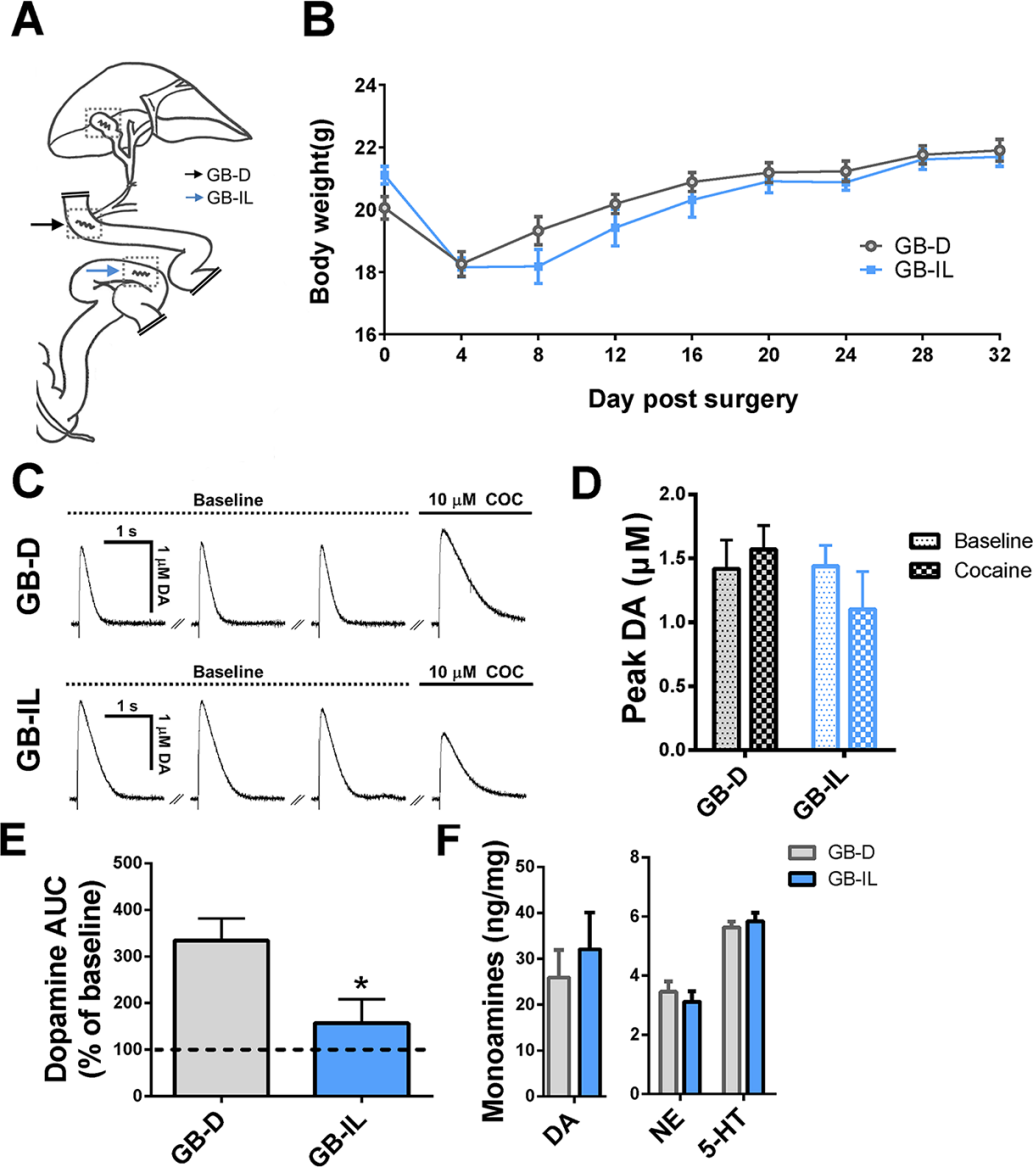
Bile diversion blocks cocaine’s ability to increase DA levels in the NAc. (**A**) Schematic representation of GB-D (black arrow) surgery and GB-IL (blue arrow) surgery. Dotted boxes outline incision sites in the gallbladder and intestine. (**B**) Body weight following GB-D or GB-IL surgery (*n* = 12–13; *p* > 0.5 by two way ANOVA). (**C**) Amperometric recordings of DA from the NAc in GB-D (top) and GB-IL (bottom) mice. Electrically evoked DA responses are stable at 5-minute intervals. Cocaine robustly enhanced the evoked DA response in the GB-D mice. This effect is blunted in GB-IL with respect to GB-D mice. (**D**) Quantitation of the peak amplitude of amperometric recordings under baseline (dotted bar) or cocaine (checkered bar) conditions in GB-D (black) or GB-IL (blue) animals (*n* = 4–5; *p* > 0.05 comparing baseline to cocaine for each surgical group, Student *t* test). (**E**) AUC of the evoked DA response in the presence of cocaine normalized to the average of the pre-cocaine baseline values from each slice (*n* = 4–5; *p < 0.05, Student *t* test). (**F**) Levels of DA, NE, and 5-HT in NAc tissue punches from GB-D and GB-IL mice. No significant differences were noted (*n* = 4–6; *p* > 0.05 by Student *t* test). Underlying data can be found in S1 Data. 5-HT, serotonin; AUC, area under the curve; DA, dopamine; GB-D, gallbladder to duodenum diversion; GB-IL, gallbladder to ileum diversion; NAc, nucleus accumbens; NE, norepinephrine.

Traditionally, bile acids have been viewed as detergents participating in the emulsification of ingested fats. It is becoming increasingly apparent, however, that bile acids also function as steroid hormones with targets in the intestine, liver, and brain [1–4]. Bile acids produced from cholesterol in the liver enter the proximal small intestine at the duodenum and are reabsorbed into hepatic portal circulation at the distal ileum, a segment of the small intestine densely populated by bile acid receptors and reuptake transporters. Bile diversion—a newly developed bariatric surgical procedure in mice—is capable of chronically elevating circulating bile acids beyond the enterohepatic bile pool through ligation of the common bile duct and anastomosis of the gallbladder to the ileum (GB-IL) (Fig 1A) [5]. In the control surgery, the gallbladder is anastomosed to the duodenum (GB-D) (Fig 1A), restoring normal bile flow as well as circulating bile acid levels.

Bile diversion was recently developed in mice to treat high-fat diet–induced obesity [5]. GB-IL mice exhibit reduced high fat food consumption as well as weight loss. This reduction in the intake of rewarding, calorically dense food could stem at least in part from altered valuation of palatable food. Reward is a process regulated, among other factors, by dopamine (DA) signaling and homeostasis. Dysregulated mesolimbic DA circuitry has been linked to augmented high-fat, high-calorie food consumption [6–8] and, importantly, to cocaine abuse [9–12]. We thus hypothesized that bile diversion to the ileum, which reduces hedonic feeding, might also reduce the rewarding properties of cocaine. Alteration in cocaine reward promoted by GB-IL would suggest a generalized mechanism by which bile acids regulate central encoding of rewards. Here, we show that GB-IL surgery is able to alter the behavioral and pharmacological responses to cocaine. This led us to uncover a novel role of central bile acid signaling mediated by Takeda G protein-coupled bile acid receptor (TGR5) for cocaine-induced impairments in DA homeostasis and the development of associated behaviors.

## Results

In GB-IL mice, there were no significant differences in body weight as compared to GB-D, up to 32 days post-surgery (Fig 1B). This strongly suggests that, on a chow diet, the long-term homeostatic regulation of body weight in GB-IL parallels that of the GB-D mice.

### GB-IL mice are resistant to cocaine-induced increase in released DA while showing no changes in total tissue DA

Cocaine directly alters DA neurotransmission and produces its rewarding effects by increasing available extracellular DA in specific brain regions, including the nucleus accumbens (NAc) [6, 13]. Behavioral pharmacological experiments indicate that increased DA transmission is clearly both necessary and sufficient to promote psychostimulant reinforcement, including the development of cocaine place preference (CPP) (for review, see Pierce and Kumaresan [14]). To evaluate whether and how GB-IL surgery regulates the reinforcing properties of cocaine, we first studied its effect on cocaine’s ability to enhance electrically evoked DA release in NAc slices. We utilized animals in the final phase of CPP (post-conditioning, day 10, see Materials and methods section). Three stable baseline recordings were taken at five-minute stimulation intervals, and no differences were noted between GB-D (Fig 1C, baseline) and GB-IL (Fig 1C, baseline) in terms of peak amperometric current (Fig 1D). Similar peak amperometric currents between the two conditions establishes that the presynaptic properties at DA synapses are unchanged by the GB-IL surgery. However, the increase in electrically evoked DA release promoted by 10 μM cocaine was significantly reduced in the GB-IL mice (Fig 1C, cocaine; quantitation in Fig 1E as area under the curve [AUC]). To further determine whether multiple exposures to cocaine are required for the GB-IL surgery to reduce cocaine’s ability to augment DA release, amperometric experiments were performed in GB-D and GB-IL animals receiving the vehicle instead of cocaine as described in Fig 1C and 1D. In cocaine-naïve animals, the GB-IL surgery significantly impaired cocaine-induced increase in DA release without altering the peak of the amperometric current (S1 Fig). These data indicate that cocaine has impaired ability to increase released DA in GB-IL mice independently of cocaine exposure.

GB-IL mice (post-conditioning, day 10) do not exhibit an overt neurochemical phenotype, as total accumbal tissue levels of DA and its related monoamines, norepinephrine (NE) and serotonin (5-HT), were not significantly altered with respect to GB-D (Fig 1F). This result further suggests that the changes in DA homeostasis promoted by cocaine are not due to the supply or release properties of DA.

### GB-IL mice show reduced expression of cocaine-induced reward-related behaviors

**Fig 2:**
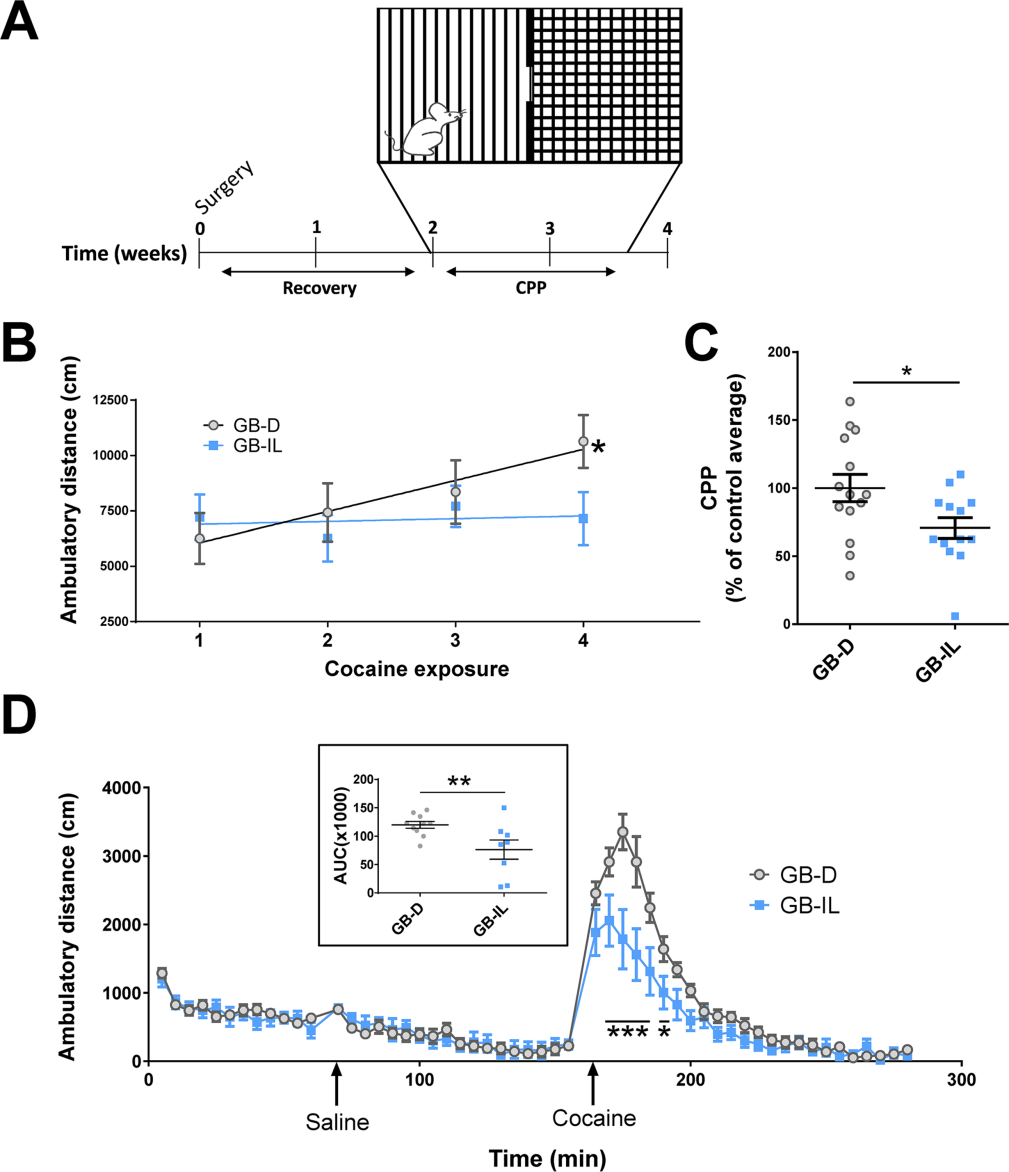
Bile diversion to the ileum blocks cocaine locomotor sensitization and reduces cocaine CPP. (**A**) Mice underwent surgery (GB-D or GB-IL), recovered for 2 weeks, and began the cocaine CPP paradigm. (**B**) Average group locomotor activity in CPP chambers during four cocaine exposures (20 mg/kg, i.p.) with linear regression of activity over the four exposures. There was a significant effect of time (Time: F[3, 69] = 2.916; *p* < 0.043), and treatment × time interaction (F[3, 69] = 2.868; *p* < 0.041) (*n* = 11–14; *p* < 0.05 by two-way repeated measuresANOVA) with a significant difference between cocaine exposure 1 and 4 in GB-D surgery animals (*p* < 0.003 by multiple comparison tests). (**C**) Cocaine CPP expressed as percent CPP normalized to GB-D average (*n* = 13–14; *p < 0.05 by Student *t* test). (**D**) OF locomotion. GB-IL mice exhibit a no difference in basal locomotion with reduced response to cocaine (*n* = 8–10; *p* < 0.05 by two-way repeated measures ANOVA, F[1, 16] = 6.544; *p < 0.05; ***p < 0.001 by multiple comparison test). (inset) AUC for cocaine locomotor responses in GB-D and GB-IL mice (**p < 0.01 by Student *t* test). Underlying data can be found in S1 Data. AUC, area under the curve; CPP, conditioned place preference; GB-D, gallbladder to duodenum diversion; GB-IL, gallbladder to ileum diversion; OF, open field.

Based on the alterations in their pharmacological response to cocaine, we next determined whether GB-IL mice display reduced behavioral responses to cocaine. Mice were tested for cocaine conditioned place preference (CPP; 20 mg/kg, intraperitoneally [i.p.]) in a dual compartment apparatus with features allowing for the animals to distinguish between the two compartments (Fig 2A). Prior to drug administration in the pre-test session, no differences in the level of cumulative baseline locomotion were observed over 30 minutes (GB-IL was 85% ± 8% of GB-D; *n* = 11–14 per group; *p* > 0.2 by Student t test). Next, one compartment of the apparatus was paired with experimenter-administered cocaine, while the second compartment was paired with experimenter-administered saline. During conditioning sessions, locomotor behavior was collected. On first exposure to cocaine, cocaine-induced hyperlocomotion was indistinguishable between the two groups. Notably, while GB-D control mice exhibited significant locomotor sensitization to cocaine over multiple exposures, the GB-IL mice did not (Fig 2B). Prior work strongly suggests that psychomotor sensitization is associated with the development of molecular adaptations within the mesocorticolimbic system in the development of an addiction [15]. The lack of locomotor sensitization in our bile diversion model may thus support impairments in the central encoding of cocaine reward. Importantly, while both groups formed a place preference for cocaine, the preference of GB-IL mice for the cocaine-paired side was significantly less than that observed for GB-D mice (Fig 2C). Four to seven days following CPP, GB-D and GB-IL mice were tested for open-field (OF) locomotion. In an OF, neither spontaneous nor saline-induced locomotion in GB-IL mice significantly differed from GB-D mice; however, cocaine-induced locomotion (20 mg/kg, i.p.) was significantly attenuated between 10 and 40 minutes post-injection (Fig 2D and inset). No change in pre-test or OF spontaneous locomotion ensures that changes in compartment preference in the CPP task were not the result of reduced locomotion in the GB-IL group. These changes in the behavioral response to cocaine are also not the result of reduced cocaine bioavailability in the striatum. We measured striatal cocaine availability in GB-D and GB-IL mice by liquid chromatography mass spectrometry (Mass Spectrometry Core, Vanderbilt University). In mice injected with cocaine (20 mg/kg, i.p.) 30 minutes prior to being euthanized we did not detect any significant difference in striatal cocaine (GB-IL was 104 ± 20% of GB-D; *n* = 9–5 per group; *p* > 0.8 by Student t test).

Furthermore, the reduction in conditioning to cocaine cannot be attributed to impaired spatial learning or memory capabilities, as we did not observe any significant impairment in performance on a hidden water maze (HWM) task (S2A–S2C Fig). No generalized impairments in motor abilities in a rotarod test (S2D Fig) or in a tail suspension test (TST) (S2E Fig) were observed. However, in the OF, we did observe a small but significant increase in center time in the GB-IL group (S2F Fig), suggesting that the surgery may also affect systems regulating exploratory behavior or anxiety.

### Bile acid signaling regulates the behavioral response to cocaine

**Fig 3:**
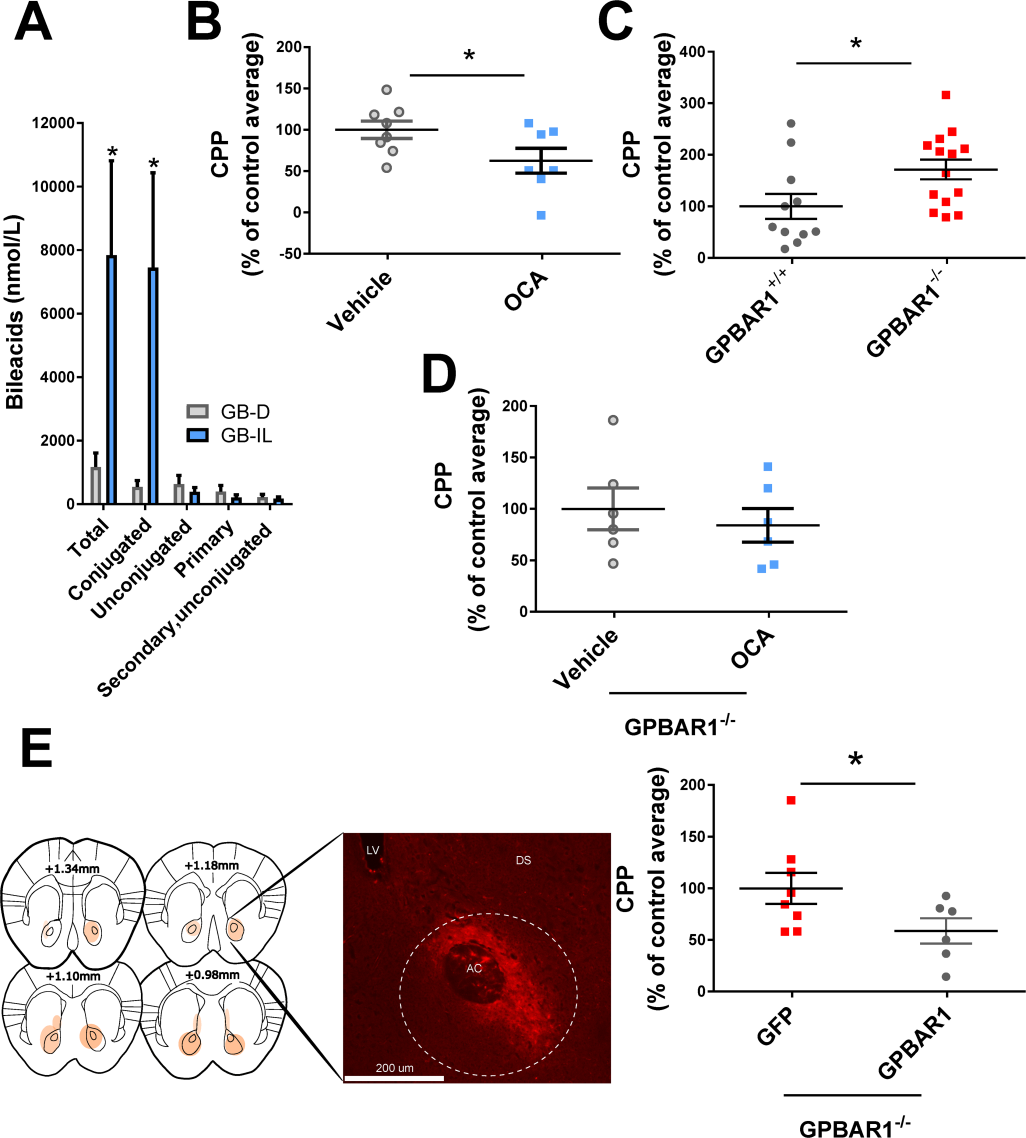
Bile acid signaling regulates cocaine reward. (**A**) GB-IL mice exhibit elevated blood levels of total and conjugated bile acids with respect to GB-D (*n* = 7–8; *p < 0.05 by Student *t* test). (**B**) OCA-treated mice showed reduced preference for the cocaine-paired chamber (*n* = 7–8; *p < 0.05 by Student *t* test) expressed as percent of CPP normalized to GB-D average. (**C**) Constitutive deletion of *Gpbar1* results in increased preference for the cocaine-paired chamber, expressed as percent of CPP normalized to *Gpbar1*^*+/+*^ littermate average (*n* = 11–14; *p < 0.05 by Student *t* test). (**D**) In *Gpbar1*^*-/-*^ mice, OCA does not impair cocaine CPP. *Gpbar1*^*-/-*^ mice do not exhibit altered cocaine CPP in response to orally administered OCA compared to vehicle controls (*n* = 6; *p* > 0.05 by Student t test). (**E**) *Gpbar1*^*-/-*^ mice virally expressing TGR5 in the NAc showed a reduced preference for the cocaine-paired chamber when compared to *Gpbar1*^*-/-*^ mice virally expressing the eGFP reporter control. Data are expressed as percent of CPP normalized to the eGFP control group average (*n* = 5–7; *p < 0.05 by Student *t* test). Underlying data can be found in S1 Data. CPP, conditioned place preference; GB-D, gallbladder to duodenum diversion; GB-IL, gallbladder to ileum diversion; Gpbar1, G protein-coupled bile acid receptor 1; NAc, nucleus accumbens; OCA, obeticholic acid; TGR5, Takeda G protein-coupled receptor 5.

Following cocaine exposure as per CPP, mice undergoing GB-IL surgery exhibit greatly increased blood levels of total and conjugated bile acids relative to GB-D, while levels of primary, secondary, and unconjugated bile acids remain unchanged (Fig 3A). Such a dramatic increase of these nutrient-signaling hormones suggests a possible role for bile acids in producing the behavioral effects of the surgery. Moreover, this elevation points to a potent and previously unexplored role for bile acid signaling as a regulator of cocaine reward, which is the focus of this study. Notably, in control animals, administration of cocaine as described in Fig 2 did not significantly alter levels of total, conjugated, or unconjugated bile acids (cocaine versus vehicle; data are expressed as percent of vehicle control; total bile acids 64 ± 11%, *p* > 0.079; conjugated bile acids 57 ± 14%, *p* > 0.079; unconjugated bile acids 99 ± 0.1%, *p* > 0.9 by Student t test, *n* = 7–8 per group). Since bile acid synthesis is regulated by the gut microbiota-to-liver axis [16], we analyzed the relative abundance and distribution of the most highly abundant resolved bacterial families in GB-IL and GB-D fecal samples. We did not find any differences in the gut microbiome composition in our surgical models (S3 Fig).

Bile acids signal as hormones mainly through two bile acid receptors: the farnesoid x receptor (*NR1H4*, FXR) and the G protein-coupled bile acid receptor 1 (GPBAR1, TGR5), which is expressed in the brain [2, 17]. Here, we show that chronic administration of the synthetic bile acid obeticholic acid (OCA), an agonist of TGR5 as well as a potent agonist of FXR [18, 19], is sufficient to reduce cocaine CPP (Fig 3B) in wild-type mice. For two weeks prior to the initiation of cocaine CPP, mice were treated orally with OCA (10 mg/kg, Per os [p.o.]) or vehicle. The treatment continued for 4 weeks following drug initiation until euthanasia. Mice treated with OCA, compared with vehicle, exhibited decreased cocaine CPP (Fig 3B).

Several conjugated and unconjugated bile acids have been found to promote phosphorylation of extracellular signal-regulated kinase (ERK) 1/2 [20]. We determined that ERK 1/2 phosphorylation measured by immunochemistry as previously described [21] was significantly elevated in the NAc of mice chronically treated as in Fig 3B with OCA (S4 Fig). These data suggest that oral administration with OCA signals at least in part in the NAc. Finally, we determined whether venous infusion of the bile acid tracer, 2,2,4,4-[2H]-taurocholic acid (d4-TCA, 0.0038 μmol·kg^-1^·min^-1^), administered at 1.5 μL/minute was capable of reaching the brain. After a 90-min tracer equilibration period, mice were anesthetized with an infusion of sodium pentobarbital, the brain was excised, immediately frozen in liquid nitrogen, and stored at −80 °C until analyzed. Mass spectrometry analysis was performed to calculate the levels of taurocholic acid-d_4_ (d4-TCA) in brain samples (50 mg). Tracer perfusion significantly increased brain d4-TCA levels 6 ± 0.1-fold with respect to vehicle treated controls (*n* = 6–7 per group; *p* < 0.01 d4-TCA versus vehicle). These data strongly suggest that altered levels of circulating bile acids in the blood correspond to parallel changes in the central nervous system (CNS).

Although cocaine acts on centrally localized targets and TGR5 is expressed in the brain [2, 17], it is possible that TGR5, FXR, or both of these receptors mediate the effect of OCA on cocaine behaviors. To discriminate between these possibilities, we first tested the involvement of TGR5 receptor signaling in the rewarding properties of cocaine by measuring cocaine CPP in TGR5 (*Gpbar1)* knockout mice (*Gbpar1*^-/-^). We found that deletion of the TGR5 receptor results in significantly increased preference for the cocaine-paired chamber relative to wild-type littermates (Fig 3C). The enhancement of cocaine reward in the *Gpbar1*^-/-^ mice identifies a role for the TGR5 receptor in reward processes and supports basal signaling through TGR5 as a contributor to resilience to cocaine reward. Furthermore, we showed that deletion of the TGR5 receptor precludes the effect of chronic OCA treatment on cocaine CPP (Fig 3D), reinforcing the importance of TGR5 in mediating the effect of OCA treatment [2, 17]. To localize the role of TGR5 in regulating cocaine behavior to the NAc, we utilized an adeno associated virus (AAV) vector to express GFP-tagged TGR5 or GFP in the NAc of *Gpbar11*^-/-^ mice and measured cocaine CPP. *Gpbar1* ^-/-^ mice virally expressing TGR5 in the NAc exhibited a significantly lower preference for the cocaine-paired chamber when compared to *Gpbar1*^-/-^ mice virally expressing GFP (Fig 3E). However, the question remains whether surgical GB-IL regulation of cocaine CPP requires TGR5 signaling. We performed GB-D and GB-IL surgeries in *Gpbar1*^*-/-*^ mice and no differences were observed in cocaine CPP (S5 Fig). These data reinforce the idea that the increase in circulating bile acids promoted by GB-IL requires TGR5 signaling in order to regulate the reinforcing properties of cocaine.

## Discussion

These findings support a role for bile acids and TGR5 signaling in neuronal function as well as in the control of motivated behaviors. This role was revealed by a novel surgery in which bile acids were diverted to the ileum to increase reabsorption and augment levels of circulating bile acids. We demonstrate that this surgery was able to modify reward acquisition and sensitization characteristic of chronic cocaine use. The GB-IL surgery blocks both sensitization and the rewarding properties of cocaine, which both rely on increases in extracellular DA levels. Notably, the surgery alters cocaine’s ability to increase DA levels in the NAc both in cocaine-naïve and cocaine-exposed animals. These results thus reveal that a surgery designed for weight loss also regulates psychostimulant reward. We found no alterations in gut microflora or striatal cocaine bioavailability in postoperative animals, making causative roles for microbiota to brain communication or reduced central cocaine concentration less likely.

In order to exploit the utility of bile diversion surgery for translational opportunities, we sought to uncover the signaling pathways mediating the effect of the surgery on cocaine induced behaviors. The main direct effect of the surgery, elevating serum bile acids, led us to consider the receptors targeted by these circulating hormones as a likely candidate mediating our observed behavioral phenotype. Consistent with this hypothesis, we demonstrate that the ability of GB-IL to inhibit cocaine CPP requires TGR5 expression. Furthermore, we show that exogenous increases in bile acid signaling through OCA administration are sufficient to reproduce the effect of the GB-IL surgery on cocaine reward–related behavior in nonsurgical animals. While OCA more potently targets FXR bile acid receptors, our results point towards a dominant role of TGR5 in mediating the effect of elevated bile acid signaling on the behavioral response to cocaine. Specifically, *Gpbar1*^*-/-*^ mice exhibit enhanced cocaine preference compared to their wild-type counterparts. Importantly, we show that OCA is incapable of altering cocaine conditioning in *Gpbar1*^*-/-*^ animals. These data suggest that TGR5 is mediating the effects of OCA and that TGR5 represents a novel target for the modulation of motivated behaviors. However, this study does not fully address the pharmacology of OCA in the brain, and future studies are required to further define bile acid signaling in the NAc. Our results add novel gut signals (bile acids) as central regulators of drug reward–related behaviors. We present evidence that TGR5 may be acting within the NAc as viral re-expression of the receptor in the NAc of *Gpbar1*^*-/-*^ mice reduces cocaine preference relative to GFP-*Gpbar1*^*-/-*^ controls. The significance of TGR5 signaling is further supported by the inability of GB-IL surgery to reduce cocaine preference in *Gpbar1*^*-/-*^ mice. Together, these results point to a role for central bile acid signaling in reducing susceptibility to cocaine and specifically implicate an accumbal receptor population. Future work could use more targeted manipulations to dissect the individual role of core and shell accumbal subregions in the effects described here. These results represent the first report of bile acids acting centrally to alter motivated behavior and open up novel avenues for translational investigations.

Thus, further studies exploring whether pharmacologic, or even surgical, enhancement of bile acid signaling could intervene in models of established addictions are warranted. Importantly, the bile acid receptor agonist used in the current study (OCA) is on the market for the treatment of primary biliary cholangitis. This drug showed clinical efficacy in this setting with an excellent safety profile, thereby reducing barriers to its application for addiction treatment. Through the identification of the bile acid signaling system as an “already drugged” target to limit cocaine reward, this work delineates a significant advancement toward novel therapies for psychostimulant addiction.

## Materials and methods

### Ethics statement

Surgical analgesia is achieved with Ketoprophen (5–10 mg/kg, subcutaneous [s.c.[, q 24 hr) at the completion of the surgical procedure and additional supplementation is provided if required. For bariatric surgical procedures, analgesia coverage will be for 72 h and 48 h, respectively, and as needed thereafter. Following all experimental procedures, the animals are euthanized with sodium Pentobarbital (125 mg/kg, intravenous [i.v.]), CO2 (inhaled), or exsanguination under anesthesia. Veterinary care and oversight is provided by the School of Medicine’s Division of Animal Care, which is staffed with 4 veterinarians. An Ethics Committee within the Division of Animal Care approved the animal experiments of this study (Protocol ID#: M/14/206). Vanderbilt University is AAALAC accredited and operates under the principles outlined in the Guide for the Care and Use of Laboratory Animals (DHEW Pub. No. (NIH) 86–23 Revised 1985).

### Mice

Male wild-type C57BL/6J mice used for surgeries or for OCA treatment were acquired from Jackson Laboratories (Bar Harbor, Maine) at 5 weeks of age. Mice were acclimated to a Vanderbilt University housing facility for one week prior to surgery. Surgery (GB-D or GB-IL) occurred at 6 weeks of age. Mice were given at least 2 weeks to recover from surgery and were handled for 3 days prior to the start of the CPP paradigm. At this point, mice either underwent behavioral testing (beginning with CPP) or were sensitized to cocaine without behavioral testing. *Gpbar1* (TGR5) knockout heterozygous breeder mice were obtained from Dr. David Wasserman and generated as described in Vassileva and colleagues [22]. Heterozygous mice were mated to generate male and female knockout mice and wild-type mice used in behavioral experiments. The temperature- and humidity-controlled facility is maintained on a 12:12 h light:dark cycle (lights on 07:00–19:00 h), and all experiments were performed during the light phase.

### Surgery

The control surgery (GB-D) and experimental surgery (GB-IL) were performed as previously described [5]. Body weights were measured immediately prior to surgery and following surgery up until sacrifice and were averaged within 4 day bins.

### Amperometry in ex vivo slice preparation

Following recovery from surgery, GB-D and GB-IL mice were treated with saline and cocaine at the dosing schedule used for cocaine CPP (briefly, i.p. injections of saline every other day for 8 days and injections of cocaine on alternate days). Mice were euthanized 1–2 days following their final cocaine injection. Nucleus accumbens slices were prepared as previously described [23]. Mice were sacrificed by rapid decapitation under isoflurane anesthesia, and 300-μm slices were prepared with a vibratome in ice-cold oxygenated (95% O2/5% CO2) sucrose solution (sucrose 210 mM; NaCl 20 mM; KCl 2.5 mM; MgCl_2_ 1 mM; NaH_2_PO_4_·H_2_O 1.2 mM; NaHCO_3_ 26 mM; dextrose 10 mM). Evoked DA release was measured in response to electrical stimulation using amperometry as described in Schmitz and colleagues [24]. Slices were maintained at 28 °C and continuously perfused with oxygenated artificial cerebrospinal fluid (ACSF) (NaCl 125 mM, KCl 2.5 mM, NaH_2_PO4·H_2_O 1.2 mM, MgCl_2_ 1 mM, CaCl_2_·2H_2_O 2 mM; NaHCO_3_ 26 mM; dextrose 10 mM). Carbon fiber electrodes were fabricated by using a 7-μm carbon fiber (Goodfellow, Coraopolis, PA) and held at a voltage of +400 mV. Recordings were performed in the NAc core at a depth of 50–75 μm in response to a single electrical pulse (200 μA, 0.1 ms) from a bipolar stimulating electrode. After stable control responses were established, 10 μM cocaine was applied to the slices.

### High-performance liquid chromatography

Mice were sacrificed by rapid decapitation under isoflurane anesthesia at 4–5 weeks following GB-D or GB-IL surgery. The brain was quickly dissected, blocked, and the NAc was punched bilaterally. Punches were stored at −80 °C until processing. To measure monoamines, high-performance liquid chromatography (HPLC) was performed as previously described.[25]

### CPP and locomotor sensitization

CPP was performed as previously described, with modifications [26]. Briefly, 2-chamber CPP apparati (MED-CPP2-MS; Med Associates, St. Albans, VT) with distinct rod and mesh floor inserts were used. The associated software allowed for automated measurement of beam breaks on X–Y–Z axes (16 infrared beams, 50-ms intervals). Mice were weighed and then acclimated to the testing room for 20 min prior to testing each day. During the first phase (pre-conditioning, day 1), mice were placed on the grid floor side of the 2-chamber apparatus. For 30 min, the mice had free access to both sides of the apparatus. During the second phase (conditioning, days 2–9), on alternate days, mice were restricted to one side or the other of the apparatus for 30 min by use of a dividing door. Just prior to being placed in the chamber, each mouse was given an i.p. injection of either cocaine (20 mg/kg) or saline. Cocaine was paired with the side of the apparatus less preferred during preconditioning. Approximately half of the mice were started on cocaine, while the other half were started on saline. During this time, each mouse’s locomotor activity was measured and used to determine cocaine-induced locomotor sensitization. The final phase of CPP (post-conditioning, day 10) consisted of placing the mouse on the cocaine-paired side initially with the dividing door removed; however, no drug was given on this day. Thus, mice were given full access to both compartments and their time spent on each side was measured. Percent of CPP was calculated as the time spent on the cocaine-paired side during post-conditioning, minus the time spent on the cocaine-paired side during pre-conditioning divided by the time spent on the saline-paired side during pre-conditioning. The first 20 min of pre-conditioning and post-conditioning were used in the calculation of percent of CPP. All CPP was performed during the first phase of the light cycle. Activity Monitor v5.10 (MED Associates) was used to analyze CPP activity.

### Mass spectrometry for cocaine bioavailability

Mice were administered cocaine (20 mg/kg, i.p) and 30 minutes later, were sacrificed by rapid decapitation under isoflurane anesthesia. The brain was quickly dissected, blocked, and the striatum was punched bilaterally with a 0.75 mm inner diameter punch. Tissue punches were placed into Eppendorf tubes on dry ice and stored at -80°C until processing.

Tissue was homogenized in 150 μL 100 mM sodium carbonate. Following centrifugation, samples were extracted using acetonitrile containing 50 nM cocaine-d_3_ as an internal standard. The resulting solution was dried under nitrogen and brought up in Mobile Phase A. Tissue cocaine content was quantified using LC-MS/MS on a Thermo TSQ Quantum ultra AM triple-quadrupole mass spectrometer in positive-ion mode using 0.1% HCOOH in Water (solvent A) and 0.1% HCOOH in Acetonitrile (solvent B). The major transition, *m/z* 304 to 182, was used to determine cocaine concentration.

### OF locomotion

Four to seven days following CPP, GB-D and GB-IL mice from selected cohorts were tested for OF locomotion. Mice were initially weighed. Following 20 min of acclimation to the testing room, mice were placed in clean automated OF chambers (28x28 cm; MED-OFA-510; MED Associates) under constant illumination for 60 min, and ambulatory distance was recorded. Mice were then removed from the chamber, injected with saline (i.p., equivalent to a 20 mg/kg dose of cocaine), and placed back in the chamber for 90 min. Finally, mice were removed again and injected with cocaine (20 mg/kg, i.p.) before being placed back in the chamber for an additional 120 min.

### Morris HWM

Following CPP and OF locomotion, GB-D and GB-IL mice from selected cohorts were tested on the HWM. The water maze protocol here was modified from a protocol previously described [27]. A round tub measuring 92 × 92 cm was filled with clean water the day before the first day of behavioral testing. On each morning of testing, mice were acclimated to the testing room for at least 10 min, after which behavioral testing began. For the first 5 days, a platform was placed just under the water in the northeast corner of the maze such that mice could not see it. Each day for 4 trials per day, mice were placed into the pool facing the wall and were given 60 seconds to find and stand on the platform. If they found it, they were allowed to stand on it for 10 seconds before being removed by the experimenter. If they did not find the platform in the 60 seconds given, they were placed on the platform by the experimenter for 20 seconds. After each trial, mice were allowed to dry in a clean cage on top of a warming pad, with at least 10 min in between each trial. On the final day of testing, the platform was removed. The mice were placed in the pool for a single trial and percentage of time in the target quadrant was measured.

### TST

Following CPP and OF locomotion, GB-D and GB-IL mice from selected cohorts were tested on the TST. This involved individually suspending each mouse by the tail using adhesive tape to a flat, stainless steel force sensor connected to a computerized monitoring system (v3.30, MED Associates). The force sensor measured the amount of time each mouse spent struggling to right itself. The mouse was suspended from the sensor for a total of 6 min. The last 4 min of the trial were used to calculate time immobile, which was defined as the total time during which the mouse movement did not exceed a preset threshold of seven for 200 ms.

### Rotarod

Following CPP and OF locomotion, GB-D and GB-IL mice from selected cohorts were tested on the rotarod. The rotarod consisted of a rotating, grooved rubber cylinder (approximately 3 cm in diameter). Mice were placed on the cylinder, which rotated for 5 min, gradually increasing from 4 to 40 rpm. The amount of time spent on the cylinder before safely falling was recorded.

### Bile acid determination

Serum bile acids were measured by mass spectrometry using methods previously described [5]. Bile acids were measured from trunk blood taken immediately following decapitation at sacrifice at 4–5 weeks and 7–8 weeks post-surgery.

### OCA administration

To allow for gut bioavailability of the semi-synthetic bile acid analogue OCA (chemically 6-ECDCA or 6α-ethyl-chenodeoxycholic acid, AdipoGen, San Diego, CA, #AG-CR1-3560-M025) without the stress of oral gavage, OCA was administered to mice by voluntary oral administration. OCA was initially dissolved in beta cyclodextrin (20% w/v) and then dissolved within palatable drug-laced jellies. Jellies were composed of gelatin (10% w/v), sucralose (18.5% w/v), artificial strawberry flavoring (8% v/v); beta cyclodextrin (2% w/v) in water. Jellies containing OCA or beta cyclodextrin without dissolved drug were made to contain 10 mg/kg based on each mouse’s original weight on the first day of drug or vehicle administration. They were given the jellies by placing each mouse into an OF chamber containing the jelly for 20–30 min on six consecutive days per week for 4 weeks. To ensure that mice consumed the jellies consistently, all mice were initially trained to eat jellies without the drug for 5 days prior to drug/vehicle jelly administration.

### Cecal content sampling and microbiota analysis

Cecal content samples were collected from GB-D and GB-IL mice at sacrifice 4–5 weeks after surgery and stored at −80 °C. Microbiota analysis was performed as previously described [5].

### Viral construct

Both the AAV2/5-*Gpbar1* (AAV2/5-CBh-m-Gpbar1-T2A-eGFP-WPRE, 3.6 × 10^12^ GC/ml) and GFP (AAV5-GFP, 1.0 × 10^13^ GC/ml) vector were obtained from Vector BioLabs (Philadelphia, PA). The AAV5-GFP solution was diluted in 5% glycerol PBS to match GC content of AAV2/5-Gpbar1.

### Viral injections

At 8 weeks of age, mice were anesthetized via isoflurane inhalation and given 0.5-μl bilateral microinjections of AAV2/5-*Gpbar1* or AAV5-GFP at a rate of 1 nL per second into the NAc (A/P + 1.3 mm, M/L +/− 1.0 mm, D/L −4.0 mm, measured from Bregma) [28]. Behavioral assays occurred three weeks after viral injection in order to coincide with the peak of AAV-mediated transgene expression [29, 30]. Following behavioral assays, animals were sacrificed and viral expression was examined. We excluded animals that did not exhibit consistent bilateral viral expression throughout the NAc.

### Brain slice preparation and immunohistochemistry

To confirm appropriate transduction and targeting of viral injections, mice were perfused with 4% paraformaldehyde in PBS and the intact brains were removed, postfixed for 24 hours, cryoprotected with 20% sucrose (PBS) overnight, and then sectioned and processed. Brains were sectioned (50 μm) using a Leica VT1000s (Buffalo Grove, IL) and stored in 0.1 M phosphate buffer. To stain, slices were permeabilized in 0.1% Triton X-100 (Thermo Fisher Scientific, Waltham, MA) in 2% goat serum (Jackson ImmunoResearch, West Grove, PA). Endogenous peroxidases were quenched with 1.0% sodium borohydride and 0.15% hydrogen peroxide (Sigma, St. Louis, MO). Slices were incubated in rabbit anti-GFP (Abcam ab290 1:2,500 at 4 °C, overnight) and HRP-conjugated goat anti-rabbit secondary antibody (Santa Cruz, CA, 1:200 at room temperature for two hours). Signal was amplified using a TSA Cyanine 3 system (Perkin Elmer, Waltham, PA).

### Statistical analysis

Data are presented as means ± standard error of the mean. Statistical analysis was performed with GraphPad Prism software, version 6 (GraphPad Software, San Diego, CA) using statistical tests noted in figure legends. Outliers were defined as having values outside of quartile 1–1.5 × interquartile range (IQR) and quartile3 + 1.5 × IQR and were excluded. A *p* value < 0.05 defined statistical significance for all tests.

## Supporting information

S1 Fig (**A**) AUC of the electrically evoked DA response in the presence of cocaine normalized to the average of the pre-cocaine baseline values from each slice in cocaine naïve GB-D (black) or GB-IL (blue). Mice had surgery at 8 weeks of age and were allowed to recover for 2 weeks prior to amperometric recording. Cocaine robustly enhanced the evoked DA response in the GB-D mice (*n* = 5–6; *p < 0.05, Student *t* test). (**B**) Quantitation of the peak amplitude of amperometric recordings under baseline (dotted bar) or cocaine (checkered bar) conditions in cocaine naïve GB-D (black) or GB-IL (blue) animals (*n* = 5–6; *p* > 0.05 comparing baseline to cocaine for each surgical group, Student *t* test). Underlying data can be found in S1 Data. AUC, area under the curve; DA, dopamine; GB-D, gallbladder to duodenum diversion; GB-IL, gallbladder to ileum diversion.
(TIF)

S2 Fig Effect of biliary diversion on performance in the Morris Water Maze, rotarod, tail suspension test, and OF center time. (**A**) There were no significant differences between GB-D and GB-IL mice in a Morris Water Maze acquisition task (*n* = 7–8; *p* > 0.05 by two-way RM ANOVA). (**B**) There were no significant differences between groups in a Morris Water Maze recall task (*p* > 0.05 by Student *t* test). (**C**) Mean swimming speed in GB-D and GB-IL mice (*p* > 0.05 by Student *t* test). (**D**) There were no significant differences in latency to fall from a rotarod (*n* = 5–8; *p* > 0.05 by two-way RM ANOVA). (**E**) Time immobile on a tail suspension task was similar between groups (*n* = 7–8; *p* > 0.05 by Student *t* test). (**F**) OF locomotion revealed a significant increase in center time in the GB-IL mice (*n* = 11–14; *p < 0.05 by Student *t* test). Underlying data can be found in S1 Data. GB-D, gallbladder to duodenum diversion; GB-IL, gallbladder to ileum diversion; OF, open field.
(TIF)

S3 Fig GB-IL mice do not exhibit altered gut microbiota compared to GB-D controls. Stacked column bar graph depicting the relative abundances and distributions of the most highly abundant resolved bacterial families across 12 fecal samples analyzed. Cecal contents from mice subject to GB-D or GB-IL were subjected to 16S RNA sequencing. Each column represents a single mouse. No significant differences in bacterial abundances were noted by a Student *t* test comparison of each bacterial family comparing grouped GB-D and GB-IL averages (*n* = 6; *p* > 0.05). Underlying data can be found in S1 Data. GB-D, gallbladder to duodenum diversion; GB-IL, gallbladder to ileum diversion.
(TIF)

S4 Fig OCA elevates pERK/total ERK ratio. (**A**) Representative immunoblots of pERK and total ERK in NAc tissue punches from mice following repeated voluntary oral administration of OCA or vehicle. Mice were given drug laced palatable noncaloric jellies in a familiar OF arena for six days a week for four consecutive weeks. Only mice that consistently consumed the entire jelly were included in the final analysis. (**B**) Quantitation of the pERK/total ERK ratio in mice treated with chronic OCA compared to vehicle control (*n* = 4; *p < 0.05 by Student *t* test). Underlying data can be found in S1 Data. NAc, nucleus accumbens; OCA, obeticholic acid; OF, open field.
(TIF)

S5 Fig GB-IL surgery does not impair cocaine CPP in Gpbar1^-/-^ mice. *Gpbar1*^*-/-*^ mice do not exhibit altered cocaine CPP in response to GB-IL surgery compared to the control surgery (*n* = 8–12 per group; *p* = 0.40 by Student *t* test). Underlying data can be found in S1 Data. CPP, conditioned place preference; GB-IL, gallbladder to ileum diversion; Gpbar1, G protein-coupled bile acid receptor 1.
(TIF)

S1 Data Data underlying Figs 1, 2 and 3 and S1–S5 Figs. (XLSX)

## Abbreviations

5-HT: serotonin
AAV: adeno associated virus
ACSF: artificial cerebrospinal fluid
AUC: area under the curve
CNS: central nervous system
CPP: conditioned place preference
d4-TCA: taurocholic acid-d_4_
DA: dopamine
eGFP: enhanced green fluorescent protein
ERK: extracellular signal-regulated kinase
FXR: farnesoid x receptor
GB-D: anastomosis of the gallbladder to the duodenum
GB-IL: anastomosis of the gallbladder to the ileum
HPLC: high-performance liquid chromatography
HWM: hidden water maze
i.p.: intraperitoneally
IQR: interquartile range
i.v.: intravenous
NAc: nucleus accumbens
NE: norepinephrine
OCA: obeticholic acid
OF: open field
p.o.: Per os (oral administration)
s.c.: subcutaneous
TGR5/GPBAR1: Takeda G protein-coupled receptor 5/G protein-coupled bile acid receptor 1
TST: tail suspension test
