# Supplementary figures and images for: Bile diversion, a bariatric surgery, and bile acid signaling reduce central cocaine reward
Source: PLoS Biol. 2018 Jul 26;16(7):e2006682. doi: 10.1371/journal.pbio.2006682 (PMC6061973; doi:10.1371/journal.pbio.2006682)

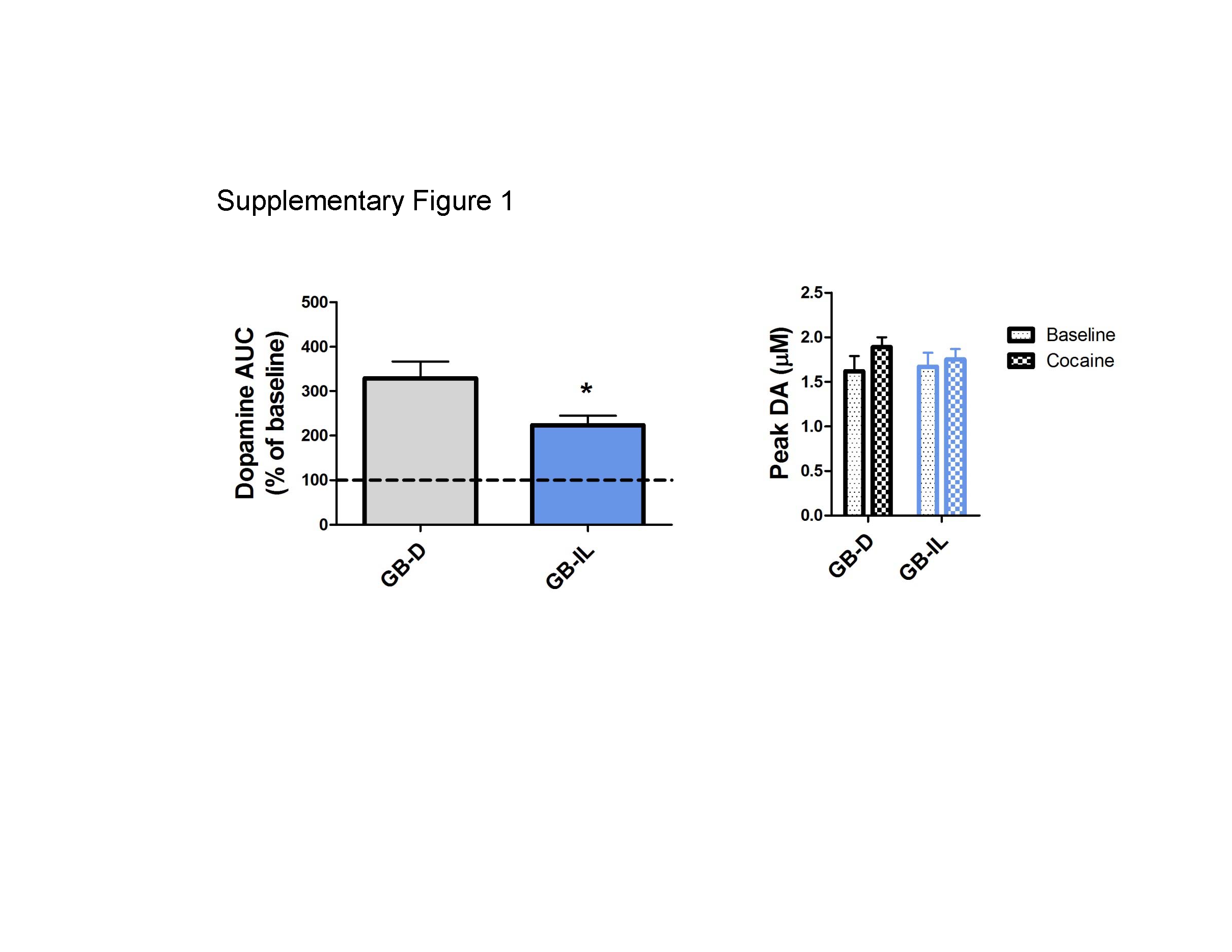

Supplement: S1 Fig — (A) AUC of the electrically evoked DA response in the presence of cocaine normalized to the average of the pre-cocaine baseline values from each slice in cocaine naïve GB-D (black) or GB-IL (blue). Mice had surgery at 8 weeks of age and were allowed to recover for 2 weeks prior to amperometric recording. Cocaine robustly enhanced the evoked DA response in the GB-D mice (n = 5–6; *p < 0.05, Student t test). (B) Quantitation of the peak amplitude of amperometric recordings under baseline (dotted bar) or cocaine (checkered bar) conditions in cocaine naïve GB-D (black) or GB-IL (blue) animals (n = 5–6; p > 0.05 comparing baseline to cocaine for each surgical group, Student t test). Underlying data can be found in S1 Data. AUC, area under the curve; DA, dopamine; GB-D, gallbladder to duodenum diversion; GB-IL, gallbladder to ileum diversion. (TIF) [file pbio.2006682.s001.tif]

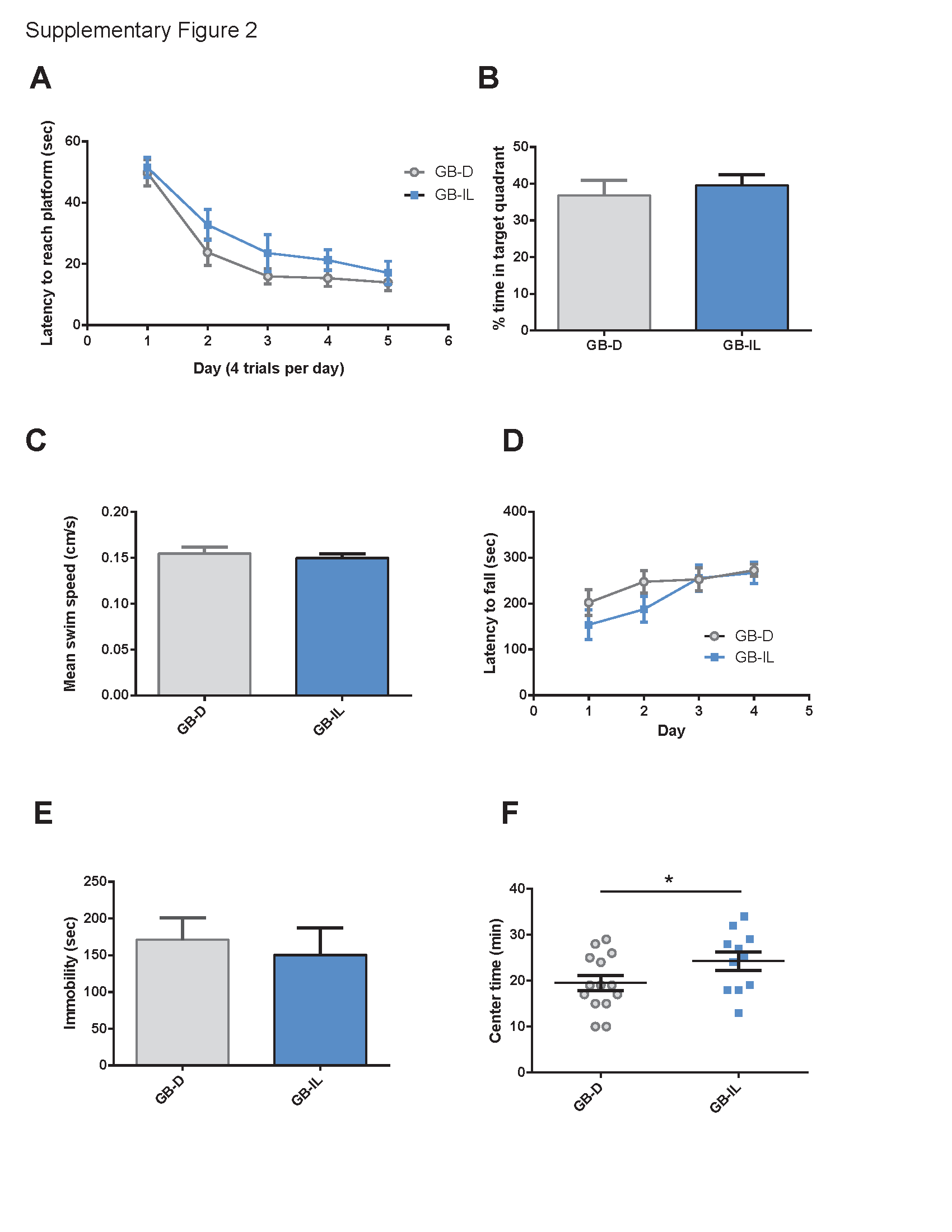

Supplement: S2 Fig — (A) There were no significant differences between GB-D and GB-IL mice in a Morris Water Maze acquisition task (n = 7–8; p > 0.05 by two-way RM ANOVA). (B) There were no significant differences between groups in a Morris Water Maze recall task (p > 0.05 by Student t test). (C) Mean swimming speed in GB-D and GB-IL mice (p > 0.05 by Student t test). (D) There were no significant differences in latency to fall from a rotarod (n = 5–8; p > 0.05 by two-way RM ANOVA). (E) Time immobile on a tail suspension task was similar between groups (n = 7–8; p > 0.05 by Student t test). (F) OF locomotion revealed a significant increase in center time in the GB-IL mice (n = 11–14; *p < 0.05 by Student t test). Underlying data can be found in S1 Data. GB-D, gallbladder to duodenum diversion; GB-IL, gallbladder to ileum diversion; OF, open field. (TIF) [file pbio.2006682.s002.tif]

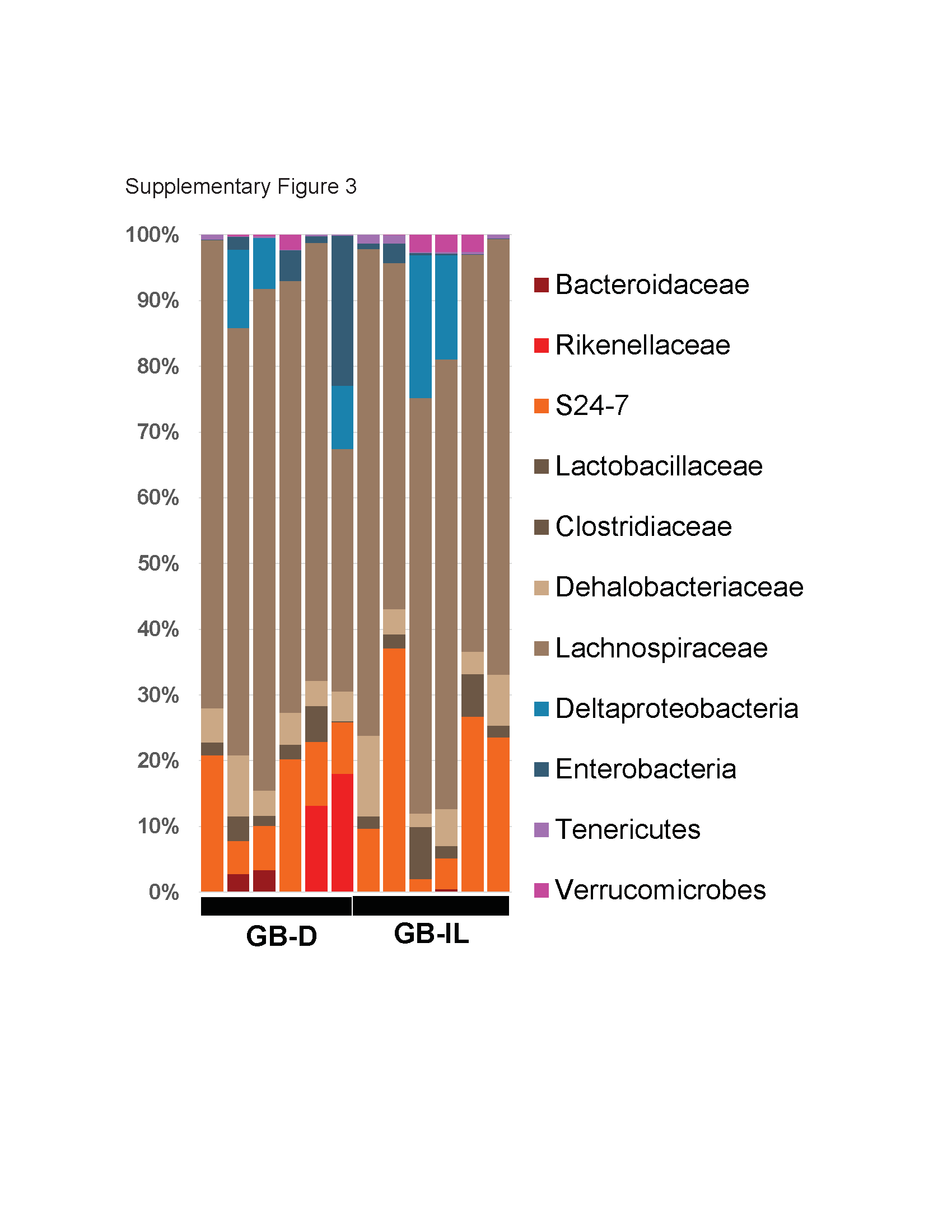

Supplement: S3 Fig — Stacked column bar graph depicting the relative abundances and distributions of the most highly abundant resolved bacterial families across 12 fecal samples analyzed. Cecal contents from mice subject to GB-D or GB-IL were subjected to 16S RNA sequencing. Each column represents a single mouse. No significant differences in bacterial abundances were noted by a Student t test comparison of each bacterial family comparing grouped GB-D and GB-IL averages (n = 6; p > 0.05). Underlying data can be found in S1 Data. GB-D, gallbladder to duodenum diversion; GB-IL, gallbladder to ileum diversion. (TIF) [file pbio.2006682.s003.tif]

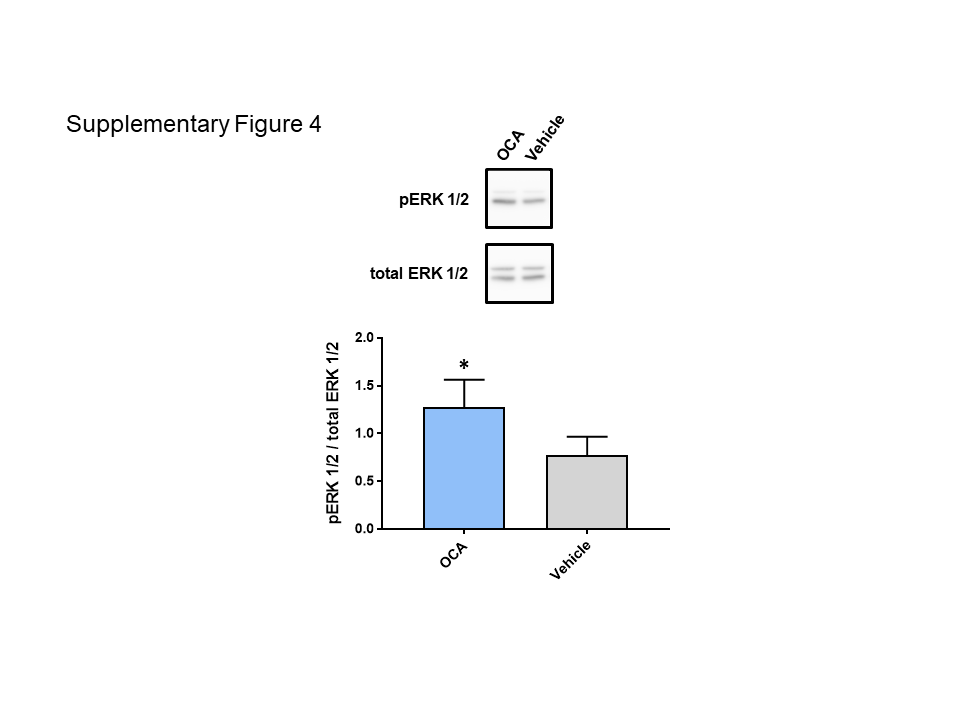

Supplement: S4 Fig — (A) Representative immunoblots of pERK and total ERK in NAc tissue punches from mice following repeated voluntary oral administration of OCA or vehicle. Mice were given drug laced palatable noncaloric jellies in a familiar OF arena for six days a week for four consecutive weeks. Only mice that consistently consumed the entire jelly were included in the final analysis. (B) Quantitation of the pERK/total ERK ratio in mice treated with chronic OCA compared to vehicle control (n = 4; *p < 0.05 by Student t test). Underlying data can be found in S1 Data. NAc, nucleus accumbens; OCA, obeticholic acid; OF, open field. (TIF) [file pbio.2006682.s004.tif]

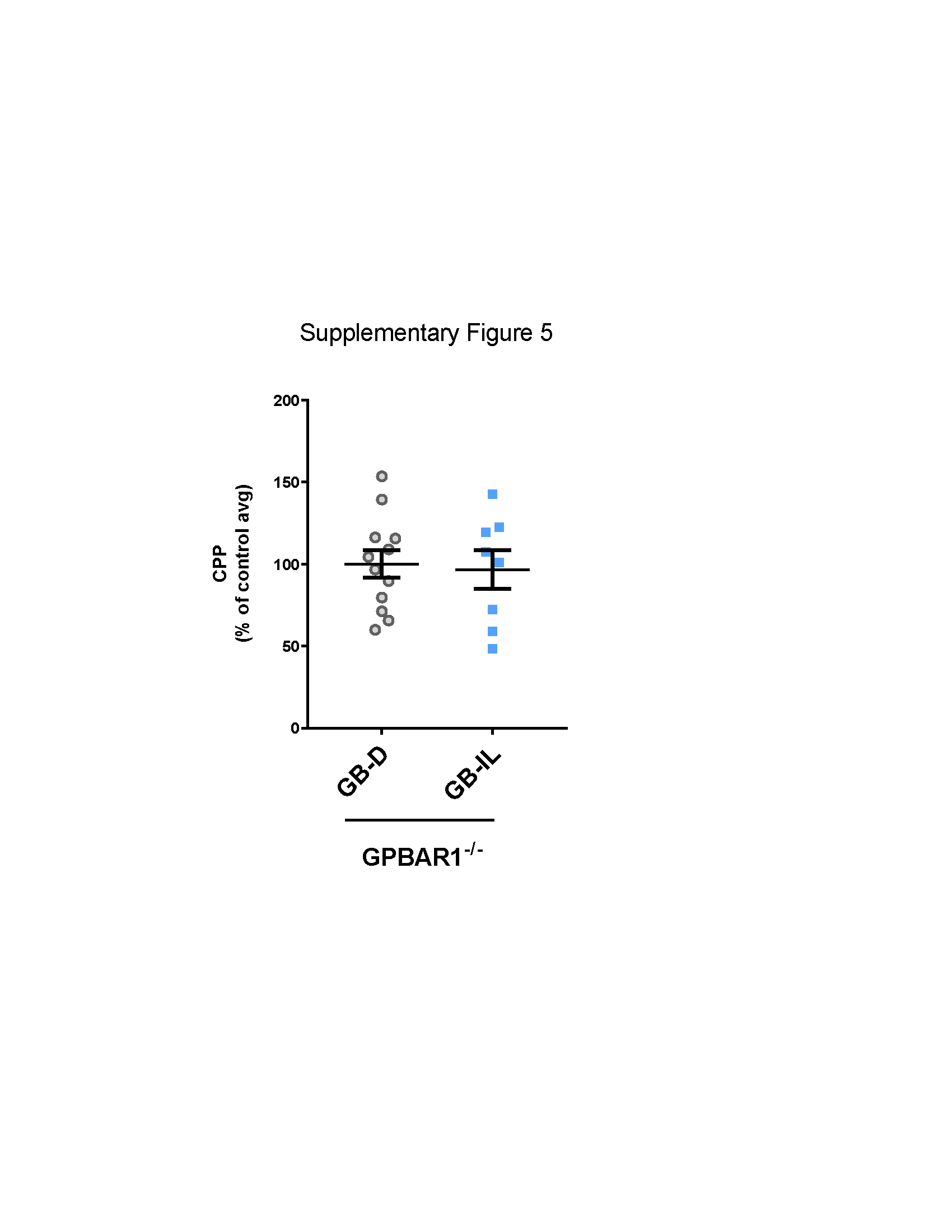

Supplement: S5 Fig — Gpbar1-/- mice do not exhibit altered cocaine CPP in response to GB-IL surgery compared to the control surgery (n = 8–12 per group; p = 0.40 by Student t test). Underlying data can be found in S1 Data. CPP, conditioned place preference; GB-IL, gallbladder to ileum diversion; Gpbar1, G protein-coupled bile acid receptor 1. (TIF) [file pbio.2006682.s005.tif]
